# Ulcerative colitis associated with chronic granulomatous disease: case report 

**Published:** 2015

**Authors:** Farid Imanzade, Aliakbar Sayarri, Pantea Tajik

**Affiliations:** *Gastroenterology Unit, Mofid Hospital, Shahid Beheshti University of Medical Sciences, Tehran, Iran*

**Keywords:** Crohn’s disease, Chronic granulomatous disease, Ulcerative colitis

## Abstract

Chronic Granulomatous Disease (CGD) is an inherited primary immunodeficiency disease which increases the body’s susceptibility to infections caused by certain bacteria and fungi. CGD is a rare disease, caused by four genes, one type is 1X linked and the other three are “autosomal recessive”. Although clinical presentation is variable, but characteristic features are recurrent pneumonia, lymphadenitis, hepatic or other abscesses. Gastrointestinal tract symptoms are common in x-linked recessive form of CGD. These include gastric and esophageal obstruction and inflammatory bowel disease. GI involvement including small and large intestines, the findings of luminal narrowing and the presence of granuloma can make it difficult to distinguish from Crohn’s disease. On the other hands according to the literature ulcerative colitis is rarely reported in patients with CGD. Our case presented with ulcerative colitis with CGD.

## Introduction

 Chronic Granulomatous Disease (CGD) is an inherited primary immunodeficiency disease which the immune system has difficulty to forming the reactive oxygen compounds (the superoxide radical) to kill certain ingested bacteria and fungi pathogens (1-3). Skin infections, pneumonia, lung abscesses, suppurative lymphadenitis, diarrhea secondary to enteritis, perianal or perirectal abscesses hepatic or splenic abscesses are the common presentation of CGD. Gastrointestinal (GI) manifestations, including gastric outlet obstruction and colitis, occur in up to 25–50% of patients. On the other hand, the prevalence and severity of GI involvement in X-linked patients was significantly higher than in autosomal recessive patients (3). Different studies suggest that GI involvement should be sought in patients who have CGD with abdominal pain, growth delay, or hypoalbuminemia (3,4). 

## Case Report

A 24-month-old male presented with abdominal pain, recurrent hematochezia, pallor, and fatigability is admitted to our hospital one month ago. For recurrent lung infections, he received several courses of antibiotics. He was admitted one year ago because of pneumonia. He was pale and on examination, his height and weight were below the third percentile.. Abdominal examination revealed generalized tenderness with no palpable masses and rectal examination revealed bloody stool. No anal fissure or hemorrhoids were seen. Hemoglobin was 5.5 mg/dl. Platelets and coagulation studies were normal. Liver and thyroid function tests were normal. NBT test for CGD was zero (0). Stool examination showed many RBC and WBC. Stool sample collected several times and cultures were negative each time (Table 1). 

**Table 1:** Biochemistry of patient at the admission time

Lab	Admission	After Treatment
WBC	18000	12500
RBC	235x10^4^	350 x10^4^
HB	5.5	10
MCV	70	76
PLT	14 x10^4^	20 x10^4^
S/E WBC RBC	Many Many	60-70 20-30
S/C	NL	NL
NBT	0	0
LFT	NL	NL
TFT	NL	NL
IG electrophoresis	NL	NL
PBS	NL	NL
ESR	90	45
CRP	4+	1+
HIV	Neg	Neg
PPD	Neg	Neg

Abdominal sonography was normal. Colonoscopy revealed erythematous and friable mucosa with multiple ulcers and areas of bleeding involving the whole mucosa of the rectum and colon up to cecum. Biopsies from colon showed prominent infiltrate of the lamina propria by lymphocytes and plasma cells intermixed with eosinophil and neutrophils. Also goblet cells depletion, occasional cystitis and early abscess formation were detected. No granulomata were seen. The inflammation was diffused and largely confined to the mucosa and these findings are consistent with ulcerative colitis. The patient was received two blood transfusions and started on pentasa, systemic steroids and cotrimoxazole. His weight, diarrhea and hemoglobin improved markedly. Unfortunately, his symptoms relapsed after tapering the systemic steroids over a period of eight weeks. This necessitated a second course of systemic steroids with slow tapering over a period of three months. 

## Discussion

Clinical presentation of CGD includes recurrent infections of the lungs, lymph nodes and skin. Bones, liver and gastrointestinal tract are less commonly involved. Infection is usually caused by catalase positive organism such as *Staphylococci*, *Pseudomonas* *aeroginosa*, *Aspergillus fumigatus*, *Candida albicans*, *Enterobacteriaceae* (*Klebsiella Serrati*) and*Mycobacterium tuberculosis* which produces a heat-labile catalase workable only at body temperatures. Residual NADPH oxidase may attenuate CGD. The majority of the infective episodes are caused by *S. aureus* and *aspergillus* (1-4). 

There may be an increased susceptibility to *Mycobacterium* including the BCG vaccine (4). Obstructive lesions of the gastrointestinal and urinary tract occur in CGD especially in the x-linked form (5). In CGD, the NBT was low (always zero) because the phagocytes cannot kill the organisms, especially *S.aureus* and *aspergillus *(5,6). Our case is most likely the x-linked form of CGD. Several histological reports of enteritis and colitis in CGD describe granulomatous lesions resembling Crohn disease (6-9). The most gastrointestinal manifestation of CGD is like the Crohn’s disease with the granulomatous formation (9). The etiology of colitis in CGD is not well understood. However, the bacteria cell wall products may act as a stimulatory factor in inflammatory bowel disease. In CGD, the persistence of viable bacteria within the phagocyte in the colonic mucosa may cause excessive stimulation of the inflammatory process and subsequent mucosa damage. Colitis associated with CGD can be intractable to the treatment (9). In some cases of ulcerative like colitis, total colectomy was done (9,10). A 12-year-old boy with CGD and colitis resembling Crohn's disease responded to treatment with cyclosporine after failure to control the colitis with high doses of corticosteroids (10). Our patient responded to the treatment with prolonged courses of corticosteroid, so we recommended to follow up these kind of patients life times. 
